# Bacteriocin-Producing *Lactiplantibacillus plantarum* YRL45 Enhances Intestinal Immunity and Regulates Gut Microbiota in Mice

**DOI:** 10.3390/nu15153437

**Published:** 2023-08-03

**Authors:** Yushan Bu, Yisuo Liu, Tai Zhang, Yinxue Liu, Zhe Zhang, Huaxi Yi

**Affiliations:** 1 College of Food Science and Engineering, Ocean University of China, Qingdao 266000, China; buyushan@stu.ouc.edu.cn (Y.B.); liuyisuo@stu.ouc.edu.cn (Y.L.); tyzhang@stu.ouc.edu.cn (T.Z.); liuyinxue_1121@126.com (Y.L.); 2 Food Laboratory of Zhongyuan, Luohe 462300, China

**Keywords:** bacteriocin-producing, *L. plantarum* YRL45, probiotics, intestinal immunity, gut microbiota

## Abstract

Bacteriocins production is one of important beneficial characteristics of probiotics, which has antibacterial property against intestinal pathogens and is helpful for regulating intestinal flora. To investigate the impact of bacteriocin-producing probiotics on gut microecology, bacteriocin-producing *Lactiplantibacillus plantarum* YRL45 was orally administered to mice. The results revealed that it promoted the release of cytokines and improved the phagocytic activity of peritoneal macrophages to activate the immune regulation system. *L. plantarum* YRL45 was conducive to maintaining the morphology of colon tissue without inflammation and increasing the ratio of villus height to crypt depth in the ileum. The gene expression levels of *Muc2*, *ZO-1* and *JAM-1* were significantly up-regulated in the ileum and colon, and the gene expression of *Cramp* presented an upward trend with *L. plantarum* YRL45 intervention. Moreover, *L. plantarum* YRL45 remarkably enhanced the levels of immunoglobulins sIgA, IgA and IgG in the intestine of mice. The 16S rRNA gene analysis suggested that *L. plantarum* YRL45 administration up-regulated the relative abundance of the beneficial bacteria *Muribaculaceae* and *Akkermansia*, down-regulated the abundance of the pathogenic bacteria *Lachnoclostridium*, and promoted the production of acetic acid, propionic acid and total short-chain fatty acids (SCFAs) in mice feces. Our findings indicated that *L. plantarum* YRL45 had the potential to be developed as a novel probiotic to regulate the intestinal barrier by altering gut microbiota to enhance intestinal immunity and ameliorate intestinal flora balance.

## 1. Introduction

Probiotics can colonize in the host intestinal tract to produce advantageous metabolites, enhance intestinal immunity and improve the balance of the intestinal flora, which have beneficial effects on the host health [1]. At present, lactic acid bacteria and *Bifidobacteria* are important sources of probiotics, which are widely used in the food and pharmaceutical industries to regulate immune function and protect the intestinal barrier, thereby improving enteritis, obesity, diabetes and other diseases [2]. It has been reported that probiotics can inhibit pathogens growth by synthesizing antibacterial substances such as bacteriocins, thereby regulating gut microbiota and maintaining host health [3,4]. Bacteriocins are active antibacterial peptides that have the advantages of safety, effective bacteriostasis and no drug resistance [5]. They can be used as natural preservatives to extend the shelf life of food, and served as potential substitutes for antibiotics in animal feed [6,7]. As new antibacterial drugs, bacteriocins can help alleviate pathogenic bacteria infection and may be exploited as an antitumor strategy [8]. Therefore, bacteriocins have become the research focus in the fields of food, agriculture and medicine.

With the increasingly close relationship among probiotics, the intestinal environment and human health, the roles of bacteriocins and bacteriocin-producing probiotics in regulating immunity and maintaining intestinal microbiota balance have been paid extensive attention. Meijerink et al. [9] found that bacteriocin-producing genes in *L. plantarum* WCFS1 were identified to regulate the immune response of host dendritic cells. Umu et al. [10] reported that lactic acid bacteria producing class II bacteriocins inhibited problematic bacteria in the gut of mice. In general, the structure of intestinal microflora at the phylum level remained almost unaffected, while some transient but favorable variations were observed at lower classification levels. According to the report of Guinane et al. [11], broad-spectrum bacteriocin bactofencin A had a delicate influence on *Bacteroides*, *Clostridium* and *Bifidibacterium* spp. It was pointed out that *Lactobacillus acidophilus* JCM 1132 and the strain possessing different bacteriocin-producing capacity had distinct influences on the intestinal microflora composition and metabolites production in mice [12]. At present, researches into the effects of bacteriocins and bacteriocin-producing probiotics on gut microecology are mostly limited to the intestinal microbiota structure, and the effects of bacteriocin-producing probiotics on intestinal health are rarely reported [13].

In previous research, bacteriocin-producing *L. plantarum* YRL45 was isolated from fermented milk in Yunnan, China, which had good gastrointestinal environment tolerance and colonization ability and showed anti-inflammatory activity in vitro [14]. In this study, the influence of *L. plantarum* YRL45 on intestinal microecology was comprehensively analyzed in vivo, which had significance for the application of bacteriocin-producing *L. plantarum* YRL45 as a probiotic.

## 2. Materials and Methods

### 2.1. Cultivation of L. plantarum YRL45

*L. plantarum* YRL45 was inoculated into MRS broth (Hopebio Technology, Qingdao, China) at a concentration of 2% (*v*/*v*), which was cultured at 37 °C for 24 h and passaged twice before use. Then, the cultivated strain was centrifuged at 6000 rpm for 10 min. The obtained deposits were washed twice with phosphate-buffered saline (PBS) and resuspended in PBS to a concentration of 1 × 10^9^ CFU/mL.

### 2.2. Design of Animal Experiments

The animal experiments were approved by Animal Ethics Committee of Ocean University of China (Permission number: SPXY2022030802). Specific pathogen free (SPF) six-week-old male C57BL/6J mice were housed at a temperature of 22 ± 2 °C and 50 ± 10% humidity in a 12 h light/dark cycle, and maintained on free access to both food and water. After adaptation for 1 week, the mice were randomly divided into 2 groups (*n* = 12 for each group): the control group (control) and *L. plantarum* YRL45 intervention group (YRL45). The mice in the control group and YRL45 group were given 200 μL PBS and bacterial suspension (1 × 10^9^ CFU/mL), respectively, for 4 consecutive weeks.

### 2.3. Determination of Physiological Indexes and Organ Coefficients

During the experiments, body weight of the mice was recorded every week, and food and water consumption were monitored every 3 days. The weight of liver, spleen and thymus after sacrifice were measured and organ coefficients were defined by the formula below:

(1) $$Organ\;coefficient(mg/g)=\frac{organ\;weight}{body\;weight}$$

### 2.4. Assay of Serum Cytokines

The eyeball blood of the mice was placed for 2 h, and the serum was obtained by centrifugation at 4000 rpm for 40 min. The concentrations of tumor necrosis factor-α (*TNF-α*), interleukin-6 (*IL-6*), interferon-γ (*IFN-γ*), *IL-10*, *IL-12* and *IL-1β* in serum were detected by ELISA kit (CalvinBio, Suzhou, China).

### 2.5. Determination of Peritoneal Macrophages Phagocytic Activity

After the mice were sacrificed, precooled RPMI-1640 medium (Solarbio, Beijing, China) was injected into the peritonea. The abdominal lavage fluids were collected and centrifuged at 1000 rpm for 10 min. The cells were resuspended in RPMI-1640 medium containing 10% fetal bovine serum (Biological Industries, Israel), cultured under an atmosphere of 5% CO_2_ at 37 °C for 24 h. The macrophages phagocytic activity was conducted by the neutral red uptake method as described by Lee et al. [15] with slight modifications. Briefly, a neutral red solution (Beyotime Biotechnology, Shanghai, China) was added to the cell culture plates and incubated for 30 min. The wells were cleaned three times with PBS before adding a lysis buffer (glacial acetic acid: ethanol = 1:1 (*v*/*v*)), which was allowed to stand overnight at 4 °C, and measured absorbance at 540 nm.

### 2.6. Analysis of Intestinal Histomorphology and Mucin Immunohistochemical

The ileum and colon of the mice were fixed in 4% paraformaldehyde (Servicebio Technology, Wuhan, China) for 24 h and embedded in paraffin. The tissues were sliced into 4 μm thick sections for hematoxylin and eosin (H&E) (Servicebio Technology, Wuhan, China) staining. The ileum and colon sections were observed with a light microscope (Nikon, Tokyo, Japan). The villus height and crypt depth of the ileum were calculated using ImageJ 1.53e. Immunohistochemical analysis was performed to analyze the expression level of MUC2 according to the description of Tong et al. [16], after which colon segments were visualized using a microscope and the positive area of MUC2 was calculated with ImageJ 1.53e.

### 2.7. Detection of Intestinal Immunoglobulins

The mice ileum and colon were homogenized in RIPA lysis buffer (Beyotime Biotechnology, Shanghai, China) and then centrifuged at 3000 rpm for 20 min. The collected supernatants were diluted with PBS, and sIgA, IgA and IgG in homogenate were quantified using ELISA kit (CalvinBio, Suzhou, China).

### 2.8. Real-Time qPCR Analysis of Intestinal Key Genes

The ileum and colon of the mice were homogenized in TRNzol Universal (Tiangen, Beijing, China) to extract total RNA, which was reverse transcribed with ReverTra Ace qPCR RT Master Mix with gDNA Remover (Toyobo, Osaka, Japan). Real-time qPCR was carried out on CFX96 RealTime PCR System (Bio-rad, Hercules, CA, USA). Gene expression was calculated as relative fold changes by the 2^−ΔΔCt^ method, and *β-actin* was used as an endogenous control. Specific primers were shown in Table 1.

### 2.9. The 16S rRNA Gene Sequencing of Gut Microorganisms

Fecal samples were collected on the last day of intragastric treatment. Genomic DNA was extracted using the Fast DNA SPIN extraction kit (MP Biomedicals, Santa Ana, CA, USA) as the template to amplify highly variable V3–V4 regions of the 16S rRNA genes. Forward primer 338F (ACTCCTACGGGAGGCAGCA) and reverse primer 806R (GGACTACHVGGGTWTCTAAT) were used for amplification. Libraries were constructed based on the TruSeq Nano DNA LT Library Prep Kit (Illumina, San Diego, CA, USA) and sequenced on the Illumina NovaSeq platform. The obtained high-quality sequences were clustered as Operational Taxonomic Units (OTUs) at 97% sequence identity using UCLUST. Diversity and taxonomic composition were analyzed by QIIME2 2019.4 software and R package. Microbial metabolic function was predicted by PICRUSt2.

### 2.10. Measurement of Feces SCFAs

The measurement of SCFAs in mice feces was carried out by gas chromatography-mass spectrometry (Agilent Technologies, Palo Alto, CA, USA) as described by Han et al. [17] with modifications. In brief, 0.1 g of fecal samples were mixed with 600 μL ultrapure water oscillated for 1 min. The suspension was acidified by 50% concentrated sulfuric acid (Sinopharm, Beijing, China), kept at room temperature for 5 min and vortexed, and then centrifuged at 5000× *g* for 10 min. The supernatants were mixed with anhydrous ether (Sinopharm, Beijing, China) at 1: 1 (*v*/*v*), vortexed for 30 s and centrifuged at 5000× *g* for 10 min. The upper ether layer was taken for further analysis. Chromatographic analysis was performed on HP–FFAP column (30 m × 250 μm × 0.25 μm; Agilent Technologies, Palo Alto, CA, USA). The procedure was set at an initial temperature of 90 °C and held for 2 min, increased to 150 °C at a rate of 12 °C/min and then increased to 220 °C at a rate of 20 °C/min and kept for 4.5 min. The running time was 15 min. Acetic, propionic and butyric acids (Macklin Biochemical, Shanghai, China) were used as standard solutions.

### 2.11. Statistical Analysis

All data were shown as mean ± standard deviation (SD). Statistical differences were analyzed by a Student’s *t*-test using IBM SPSS Statistics 22.0; *p* < 0.05 was considered statistically significant.

## 3. Results and Discussion

### 3.1. Bacteriocin-Producing L. plantarum YRL45 Had No Adverse Effect on Physiological Indexes and Organ Coefficients of Mice

*L. plantarum* YRL45 with probiotic potential in vitro was administered to mice via oral gavage. It was shown that *L. plantarum* YRL45 intervention did not significantly change the body weight, food intake or water consumption throughout the experiment (*p* > 0.05) (Table 2), indicating that there was no obvious adverse effect from bacteriocin-producing *L. plantarum* YRL45 to mice in the short term. The results of organ coefficients revealed that *L. plantarum* YRL45 supplementation had no significant effect on the liver or spleen coefficients (*p* > 0.05), while an insignificant increasing trend was found for the thymus coefficient (*p* = 0.061) (Table 3). The thymus is a pivotal organ for immune system function, because it controls and coordinates immune system development [18]. After intragastric administration of *L. plantarum* YRL45, this increasing trend demonstrated that *L. plantarum* YRL45 might have a certain regulatory role in the immune system of mice, but further explorations in combination with various immune-related indicators were required to elucidate the immunoregulatory function of *L. plantarum* YRL45.

### 3.2. Bacteriocin-Producing L. plantarum YRL45 Activated the Immune Regulatory System

**Figure 1:**
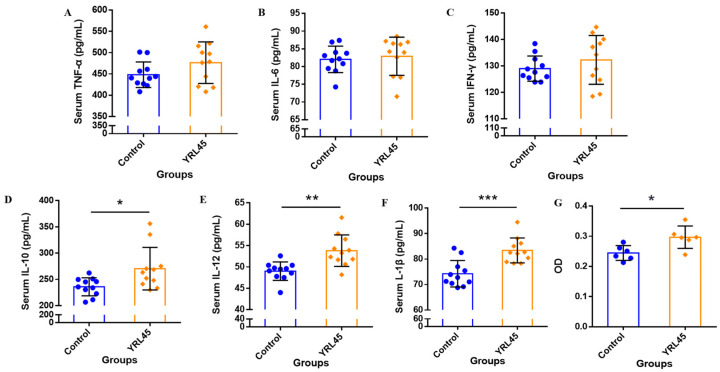
Effect of *L. plantarum* YRL45 on the secretion of cytokines in serum (*n* = 11) and peritoneal macrophages phagocytic activity of mice (*n* = 6). (**A**) The content of *TNF-α* in serum. (**B**) The content of *IL-6* in serum. (**C**) The content of *IFN-γ* in serum. (**D**) The content of *IL-10* in serum. (**E**) The content of *IL-12* in serum. (**F**) The content of *IL-1β* in serum. (**G**) The phagocytic activity of peritoneal macrophages. * *p* < 0.05, ** *p* < 0.01, *** *p* < 0.001.

The secretion of cytokines is closely related to immune function, and macrophages are crucial for the regulation of body immune. To further explore the influence of bacteriocin-producing *L. plantarum* YRL45 on the immune system of mice, the contents of six common cytokines in serum and the phagocytic activity of peritoneal macrophages were determined. It turned out that *L. plantarum* YRL45 had no remarkable influence on the levels of *TNF-α*, *IL-6* and *IFN-γ* in serum of mice (*p* > 0.05), but the concentrations of *IL-10* (*p* < 0.05), *IL-12* (*p* < 0.01) and *IL-1β* (*p* < 0.001) increased significantly (Figure 1A–F). It can be seen from Figure 1G that *L. plantarum* YRL45 increased the peritoneal macrophages phagocytic activity remarkably (*p* < 0.05). The above data showed that *L. plantarum* YRL45 stimulated the phagocytosis of macrophages, and promoted the production of cytokines, which was beneficial for boosting the immune system and achieving immune regulation in an immunosuppressive state. The changes in the levels of pro- and anti-inflammatory cytokines maintained homeostasis in the host and balanced the inflammatory response. Relevant studies have shown that bacteriocins enhanced immune regulation by promoting macrophages phagocytosis and cytokines production. Wang et al. [19] found that the bacteriocin sublancin from *Bacillus subtilis* regulated innate immunity by inducing the release of *IL-1β*, *IL-6*, *TNF-α* and nitric oxide and improving the phagocytic activity of mice peritoneal macrophages, which helped the host defend against bacterial infection. Another research demonstrated that the phagocytosis of peritoneal macrophages and the gene expression of *IL-1β*, *IL-6* and *TNF-α* were notably increased by oral administration of sublancin in normal mice. In cyclophosphamide-treated mice, sublancin elevated the macrophages phagocytic activity and restored the gene expression of *IL-2*, *IL-4* and *IL-6*, which could be a potential candidate for prevention against immunosuppression [20]. Our results were consistent with the above researches. It was meant that intragastric administration of *L. plantarum* YRL45 would stimulate the innate immune system by activating macrophages to produce cytokines, which was propitious to cope with body potential infection, enhance immune protection and promote host recovery. Furthermore, the influence of *L. plantarum* YRL45 on intestinal immune function would be studied.

### 3.3. Bacteriocin-Producing L. plantarum YRL45 Protected the Intestinal Mucous Layer

**Figure 2:**
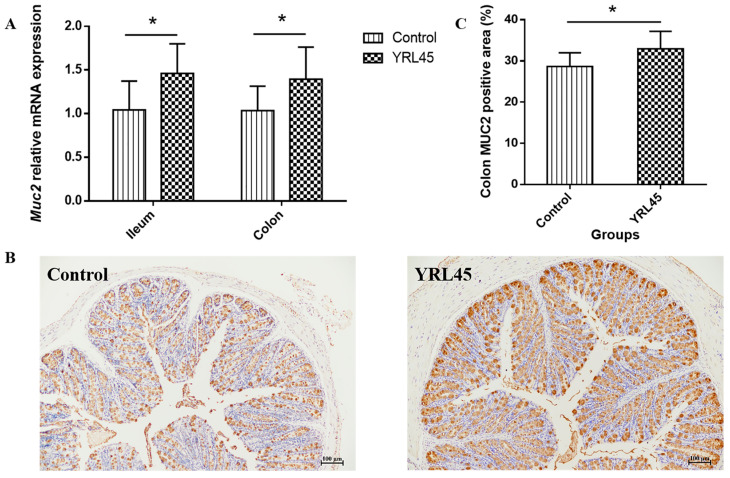
Effect of *L. plantarum* YRL45 on the intestinal mucin expression of mice (*n* = 4). (**A**) The gene expression of *Muc2* in ileum and colon. (**B**) Immunohistochemistry of MUC2 in colon. (**C**) The positive area of MUC2 in colon. * *p* < 0.05.

The main component of intestinal mucus is the mucin MUC2 secreted by goblet cells. It plays an essential part in preventing pathogenic bacteria infection and inflammation [21]. The gene expression levels of *Muc2* in the ileum and colon of mice were determined, and Figure 2A illustrated that oral administration of *L. plantarum* YRL45 significantly up-regulated the expression of *Muc2* (*p* < 0.05). Through an immunohistochemical analysis, it was found that the positive area of MUC2 in the colon was increased markedly in the YRL45 group (*p* < 0.05) (Figure 2B,C). The bacteriocin-producing *L. plantarum* YRL45 promoted the expression of intestinal mucin MUC2 in the mice, which might be favorable for protecting the intestinal mucosa from pathogens and exogenous harmful substances and boosting the lubrication and protection function of the mucus layer.

### 3.4. Bacteriocin-Producing L. plantarum YRL45 Improved the Intestinal Tissue Morphology

**Figure 3:**
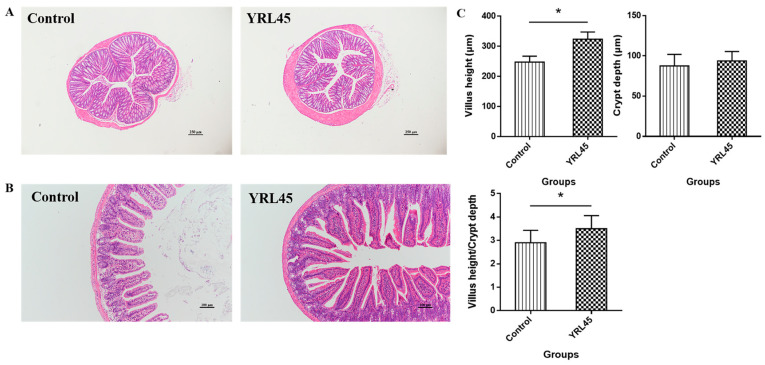
Effect of *L. plantarum* YRL45 on the intestinal tissue morphology of mice (*n* = 5). (**A**) Colon histomorphology. (**B**) Ileum histomorphology. (**C**) Villus height and crypt depth of ileum. * *p* < 0.05.

The effect of *L. plantarum* YRL45 on colon and ileum morphology were observed by H&E staining. The results in Figure 3A revealed that, compared to the control group, no obvious pathological change was found in the colon tissue of mice treated with *L. plantarum* YRL45, and the intestinal villi were intact and neat, which suggested that *L. plantarum* YRL45 did not affect the normal colon morphology. The villus height and crypt depth were used to a certain extent to evaluate the integrity of the ileum tissue structure. The increase in villus height was helpful for enhancing the absorption capacity of nutrients, while the rise of crypt depth was unfavorable for nutrition absorption. The ratio of villus height to crypt depth is an important index that reflects the function of small intestine [22]. *L. plantarum* YRL45 intervention increased the villus height of ileum remarkably (*p* < 0.05), but had no significant effect on crypt depth (*p* > 0.05) (Figure 3B,C). The ratio of villus height to crypt depth increased significantly in mice gavaged with bacteriocin-producing *L. plantarum* YRL45 (*p* < 0.05) (Figure 3B,C), which was instrumental in improving the absorption of nutrients in the small intestine and keeping intestinal structure integrity. In particular, no inflammatory change was observed in the ileum or colon, which meant that intragastric administration of *L. plantarum* YRL45 stimulated the innate immune system without leading to substantial inflammation in the intestine of healthy mice, thus maintaining the body in a balanced state.

### 3.5. Bacteriocin-Producing L. plantarum YRL45 Revealed Tight Junction Intestinal Barrier Function

**Figure 4:**
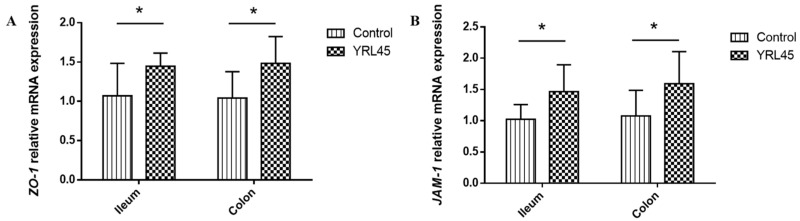
Effect of *L. plantarum* YRL45 on the intestinal tight junction proteins gene expression of mice (*n* = 4). (**A**) The gene expression of *ZO-1* in ileum and colon. (**B**) The gene expression of *JAM-1* in ileum and colon. * *p* < 0.05.

Tight junction proteins are crucial for sustaining the epithelial barrier function and controlling paracellular permeability [23]. To explore whether bacteriocin-producing probiotics regulate the intestinal tight junction system, the gene expression of the tight junction proteins *ZO-1* and *JAM-1* in the ileum and colon of the mice were detected. It turned out that *L. plantarum* YRL45 increased expression of *ZO-1* and *JAM-1* (*p* < 0.05) (Figure 4), revealing that *L. plantarum* YRL45 played a critical role in enhancing tight junction intestinal barrier function. Similarly, Heeney et al. [13] found a remarkably higher level of ZO-1 in the ileum of mice given high fat diet following administration of bacteriocin-producing *L. plantarum*. This was not the case for the mutant strain that had the bacteriocin synthesis gene knocked out, which demonstrated the necessity of bacteriocin production capacity for *L. plantarum* to protect the intestinal barrier.

### 3.6. Bacteriocin-Producing L. plantarum YRL45 Promoted the Release of Immunoglobulins in Intestine

**Figure 5:**
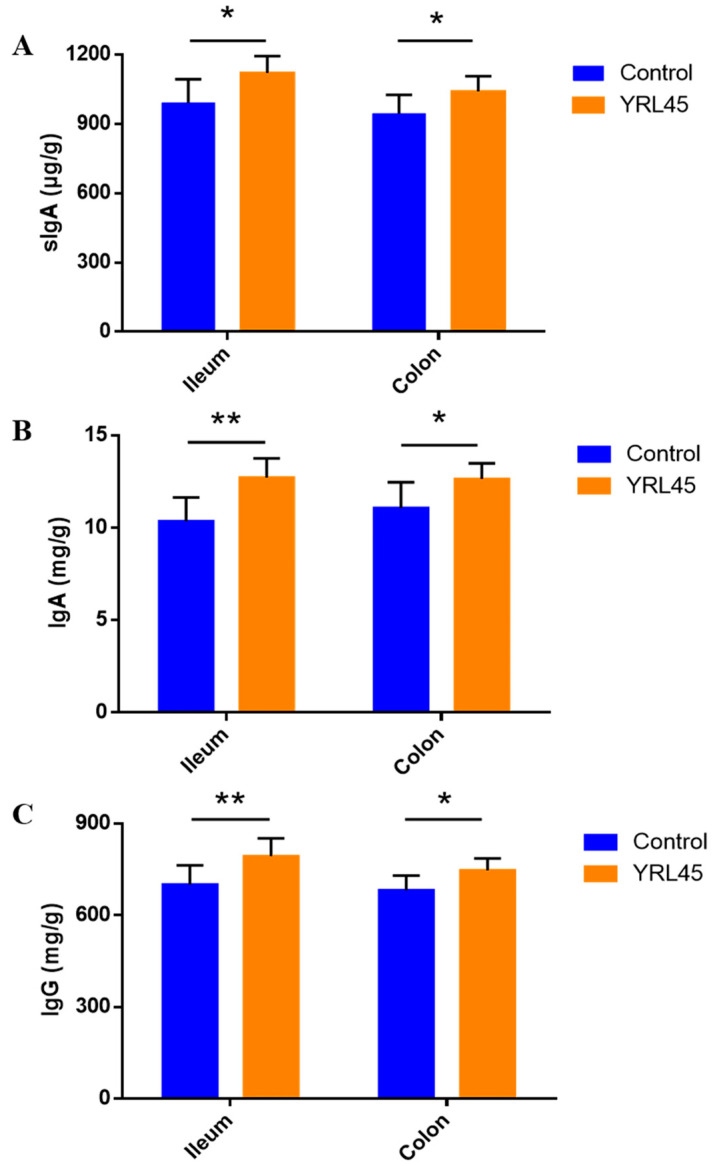
Effect of *L. plantarum* YRL45 on the secretion of immunoglobulins in intestine of mice (*n* = 8). (**A**) The contents of sIgA in ileum and colon. (**B**) The contents of IgA in ileum and colon. (**C**) The contents of IgG in ileum and colon. * *p* < 0.05, ** *p* < 0.01.

Immunoglobulins are important immune active molecules that play a significant role in enhancing the organism’s immunity and improving defense capability [24]. Changes in the secretory immunoglobulin sIgA in the intestine of mice were measured after strain intervention. Figure 5A showed that the levels of sIgA in the ileum and colon were notably increased in the YRL45 group (*p* < 0.05). The up-regulation of intestine sIgA helped inhibit pathogenic bacteria from invading the gut mucosal surface and blocked infection to body, thus protecting the integrity of the mucosal barrier and strengthening the immune function of intestine. The contents of IgA and IgG in the mice intestine were detected, and it could be seen that the administration of *L. plantarum* YRL45 led to increased IgA and IgG in the ileum (*p* < 0.01) and colon (*p* < 0.05) (Figure 5B,C), which contributed to anti-infection and anti-inflammatory functions. Bacteriocin-producing *L. plantarum* YRL45 promoted the secretion of sIgA, IgA and IgG in the intestine, which was of great significance for enhancing intestinal immune function.

### 3.7. Bacteriocin-Producing L. plantarum YRL45 Regulated Intestinal Endogenous Antimicrobial Peptide

**Figure 6:**
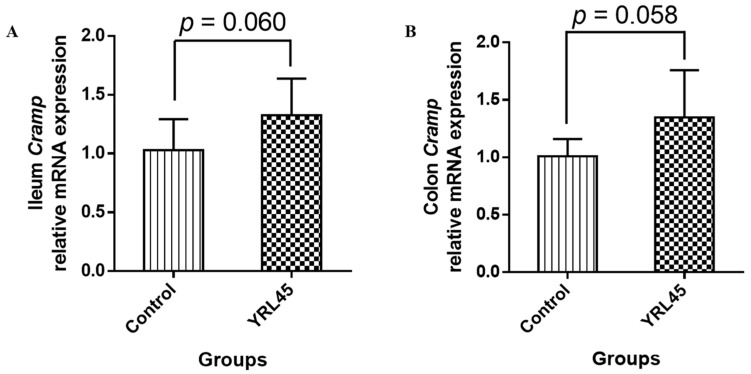
Effect of *L. plantarum* YRL45 on the intestinal endogenous antimicrobial peptide gene expression of mice (*n* = 4). (**A**) The gene expression of *Cramp* in ileum. (**B**) The gene expression of *Cramp* in colon.

As the crucial part of an innate immune defense system, antimicrobial peptides improve the non-specific immunity of body and resist infection and inflammatory reaction [25]. Cathelicidin (CRAMP) is an important antimicrobial peptide in mice produced by epithelial and immune cells [26]. As evident from Figure 6, after treatment with *L. plantarum* YRL45, the gene expression of *Cramp* in the ileum (*p* = 0.060) and colon (*p* = 0.058) showed an upward trend, so *L. plantarum* YRL45 revealed the potential for inducing the expression of endogenous antimicrobial peptides. A previous study pointed out that *Cramp* knockout mice induced by dextran sulfate sodium developed more severe colitis than wild-type mice, and cathelicidin administration reduced the severity of colitis, which suggested that cathelicidin could protect against the induction of colitis [27]. It was also found that the increasing colonic CRAMP expression in newborn non-obese diabetic mice modulated the intestinal flora and restored gut homeostasis [28]. Therefore, bacteriocin-producing *L. plantarum* YRL45 might enhance innate immunity by inducing the expression of endogenous antimicrobial peptides. This would be helpful for host defense and damage repair and be conducive to maintaining intestinal homeostasis. It is worth mentioning that further validation of immunomodulatory function of *L. plantarum* YRL45 in the disease model is still needed.

### 3.8. Bacteriocin-Producing L. plantarum YRL45 Regulated Intestinal Microorganisms

**Figure 7:**
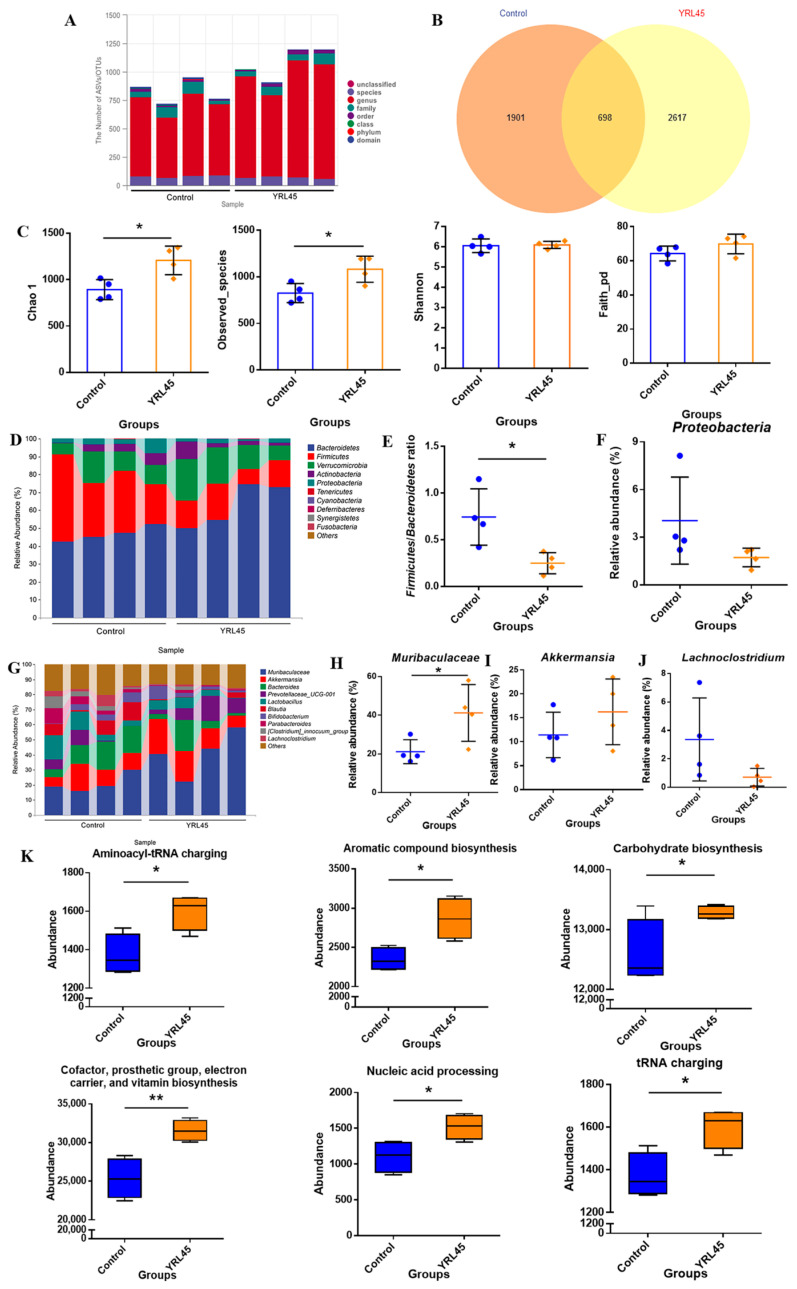
Effect of *L. plantarum* YRL45 on intestinal microorganisms of mice (*n* = 4). (**A**) Taxonomic annotation of species. (**B**) Venn diagram of ASVs/OUTs. (**C**) Alpha diversity indexes. (**D**) Bar graphs of species composition at phylum level. (**E**) The ratio of *Firmicutes* to *Bacteroidetes*. (**F**) The relative abundance of *Proteobacteria*. (**G**) Bar graphs of species composition at genus level. (**H**) The relative abundance of *Muribaculaceae*. (**I**) The relative abundance of *Akkermansia*. (**J**) The relative abundance of *Lachnoclostridium*. (**K**) The statistics of KEGG metabolic pathway. * *p* < 0.05, ** *p* < 0.01.

To clarify the influence of *L. plantarum* YRL45 on mice intestinal flora is essential for exploring the function of bacteriocin-producing probiotics on regulating the intestinal barrier. Feces from mice were collected for 16S rRNA gene sequencing and the data showed that the number of OTUs in the YRL45 group was higher than that in the control group (Figure 7A,B). The results of α-diversity of intestinal flora were revealed in Figure 7C. *L. plantarum* YRL45 intervention notably increased Chao 1 and Observed_species (*p* < 0.05), and there was no remarkable change in Shannon and Faith_pd (*p* > 0.05), indicating that *L. plantarum* YRL45 led to an increase in the richness of microbial communities, but no remarkable difference in diversity or uniformity was found.

The species composition of intestinal flora in mice was further analyzed. The relative bacterial abundance at the phylum level showed that the ratio of *Firmicutes* to *Bacteroides* (F/B) was significantly reduced due to the intervention of *L. plantarum* YRL45 (*p* < 0.05) (Figure 7D,E). It was reported that obese individuals showed an elevated F/B, resulting in increased energy absorption by the host [29]. Qiao et al. [4] pointed out that *Pediococcus acidilactici* CCFM28 intervention significantly reduced F/B in the gut microflora of normal mice, which might be beneficial for the host’s metabolic balance and intestinal homeostasis. In our study, the effect of *L. plantarum* YRL45 on F/B in the intestine of mice was similar to that of *P. acidilactici* CCFM28 reported previously, revealing that *L. plantarum* YRL45 had the potential to regulate body metabolism. *Proteobacteria* includes a variety of pathogens that are responsible for bacterial translocation and secondary infections [30]. As illustrated in Figure 7F, the relative abundance of *Proteobacteria* in intestinal microorganisms was decreased from 4.04% in the control group to 1.72% in the YRL45 group (*p* > 0.05). The decrease in the relative abundance of *Proteobacteria* with *L. plantarum* YRL45 treatment might be attributed to the ability of *L. plantarum* YRL45 to synthesize bacteriocin. The bacteriocin-producing *L. plantarum* YRL45 exerted antibacterial activity to inhibit the growth of intestinal pathogens.

The relative bacterial abundance at the genus level was analyzed to investigate the impact of bacteriocin-producing *L. plantarum* on the abundance of specific genera (Figure 7G). *L. plantarum* YRL45 supplementation notably increased the abundance of longevity-linked *Muribaculaceae* in feces (*p* < 0.05) (Figure 7H) [31]. It was reported that *Muribaculaceae* correlated negatively with obesity [32], so regulating the abundance of *Muribaculaceae* in the intestine by *L. plantarum* YRL45 was beneficial for alleviating adipose tissue inflammation and ameliorating lipid metabolic disorders. When *L. plantarum* YRL45 was administered, the relative abundance of the promising probiotic *Akkermansia* in the gut was increased from 11.43% to 16.25% (*p* > 0.05) (Figure 7I), and the rise of *Akkermansia* abundance was important for exerting anti-inflammatory effect and improving the host metabolic function and immune response [33]. *Lachnoclostridium* was a representative enterogenous pathogen, which was significantly enriched in colorectal cancer patients [34]. The relative abundance of *Lachnoclostridium* in the control group was 3.37% and decreased to 0.72% after *L. plantarum* YRL45 intervention (*p* > 0.05) (Figure 7J), which was helpful for inhibiting pathogens growth and promoting intestinal health.

Statistical analysis of the KEGG metabolic pathway was carried out for mice intestinal flora. It can be seen from Figure 7K that *L. plantarum* YRL45 had a significant impact on the functions of intestinal microorganisms in aminoacyl-tRNA charging (*p* < 0.05), aromatic compound biosynthesis (*p* < 0.05), carbohydrate biosynthesis (*p* < 0.05), cofactor, prosthetic group, electron carrier, and vitamin biosynthesis (*p* < 0.01), nucleic acid processing (*p* < 0.05), and tRNA charging (*p* < 0.05), which showed that *L. plantarum* YRL45 modulated the transcription, translation and biosynthesis of intestinal microflora and stimulated body growth and metabolism.

### 3.9. Bacteriocin-Producing L. plantarum YRL45 Favored the Production of SCFAs

**Figure 8:**
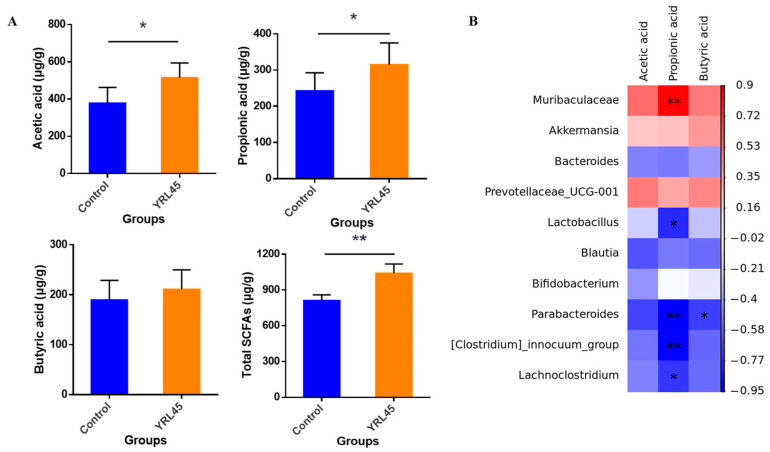
Effect of *L. plantarum* YRL45 on SCFAs in feces of mice (*n* = 6). (**A**) The contents of acetic acid, propionic acid, butyric acid and total SCFAs in feces. (**B**) The association heatmap of gut microbiota and SCFAs. * *p* < 0.05, ** *p* < 0.01.

SCFAs are major metabolites of intestinal flora and crucial energy sources for colon and ileum cells [35]. Based on the influence of *L. plantarum* YRL45 on the intestinal microflora of mice, the changes of SCFAs were further determined. The results showed that oral administration of *L. plantarum* YRL45 significantly up-regulated the levels of acetic acid (*p* < 0.05), propionic acid (*p* < 0.05) and total fecal SCFAs (*p* < 0.01) (Figure 8A), which provided energy sources for the host and displayed the anti-inflammatory capacity. An analysis between intestinal flora and SCFAs showed that *Muribaculaceae* and *Akkermansia* were correlated positively with acetic acid, propionic acid and butyric acid, whereas *Lachnoclostridium* had a negative correlation (Figure 8B). Zhao et al. [36] pointed out that propionic acid abundance was correlated positively with *Muribaculaceae* and negatively with inflammatory markers. Kim et al. [37] reported that the contents of acetic acid and propionic acid in the intestine of mice were increased notably after intragastric administration of *Akkermansia*. It was also found that there was a negative correlation between *Lachnoclostridium* and SCFAs [38]. Our findings were in line with those of the studies mentioned above. *L. plantarum* YRL45 up-regulated the abundance of *Muribaculaceae* and *Akkermansia*, down-regulated the abundance of *Lachnoclostridium*, and markedly increased the contents of acetic acid and propionic acid, which indicated that an increase in beneficial bacteria and decrease in pathogens could promote SCFAs production.

### 3.10. The Correlation of Bacteriocin-Producing L. plantarum YRL45, Gut Microbiota, and Intestinal Key Genes

**Figure 9:**
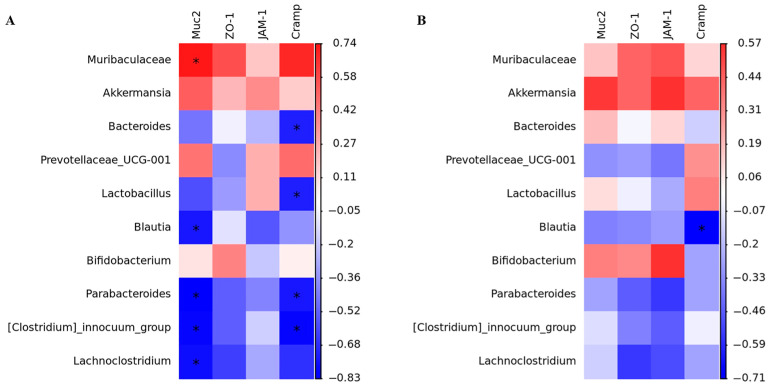
Correlation heatmap of gut microbiota and intestinal key genes in mice. (**A**) Correlation heatmap of gut microbiota and intestinal key genes in ileum. (**B**) Correlation heatmap of gut microbiota and intestinal key genes in colon. * *p* < 0.05.

The association of intestinal microbes and four key intestinal genes in mice gavaged with *L. plantarum* YRL45 was conducted. In Figure 9, *Muribaculaceae* and *Akkermansia* were correlated positively with the gene expression of *Muc2*, *ZO-1*, *JAM-1* and *Cramp* in the ileum and colon of mice, while *Parabacteroides* and *Lachnoclostridium* were correlated negatively. Similarly, Schneider et al. [39] reported that the expression of ZO-1 was increased in the gut of mice fed *Akkermansia*. Previous research demonstrated that a negative correlation between *Lachnoclostridium* and *Muc2* expression was observed [38]. It was pointed out that *Parabacteroides* abundance was correlated negatively with the level of *ZO-1*, which could affect the intestinal barrier [40]. Therefore, it was concluded that bacteriocin-producing *L. plantarum* YRL45 regulated the gene expression of intestinal mucin, tight junction proteins and endogenous antimicrobial peptide by affecting intestinal flora, thereby enhancing intestinal immunity, improving intestinal microecological balance and promoting body health.

## 4. Conclusions

In this study, the influence of bacteriocin-producing *L. plantarum* YRL45 on gut microecology in mice was investigated. *L. plantarum* YRL45 demonstrated immunomodulatory abilities in increasing cytokine levels and peritoneal macrophages phagocytic activity. In addition, the strain was conductive to promoting intestinal mucin expression, improving intestinal tissue morphology, and exerting tight junction intestinal barrier function, which also promoted the production of immunoglobulins and stimulated the expression of endogenous antimicrobial peptide in the intestine. The regulation mechanism of *L. plantarum* YRL45 on the gut might be associated with promoting favorable changes in intestinal flora and the production of SCFAs. Our findings highlighted that bacteriocin-producing *L. plantarum* YRL45 enhanced intestinal immunity and modulated gut microbiota, which could be considered a valuable probiotic for ameliorating intestinal microecology and facilitating gut health.

## Figures and Tables

**Table 1:** Primers for Real-time qPCR.

Gene Names	Primer Sequences (5′-3′)
*β-actin*	F: GTGCTATGTTGCTCTAGACTTCG
	R: ATGCCACAGGATTCCATACC
*Muc2*	F: TGCTGACGAGTGGTTGGTGAATG
	R: TGATGAGGTGGCAGACAGGAGAC
*ZO-1*	F: GCTGCCTCGAACCTCTACTC
	R: TTGCTCATAACTTCGCGGGT
*JAM-1*	F: AGTTCGTCCAAGGCAGCACAAC
	R: AGAAGGTGACTCGGTCCGCATAG
*Cramp*	F: GTCACTATCACTGCTGCTGCTACTG
	R: GATCCAGGTCCAGGAGACGGTAG

**Table 2:** Effect of *L. plantarum* YRL45 on body weight and food and water intake of mice.

Groups	Body Weight (g)				Food Intake (g/Mouse/3 Days)	Water Intake (mL/Mouse/3 Days)
	First Week	Second Week	Third Week	Fourth Week		
Control	22.89 ± 0.70	23.67 ± 0.66	24.56 ± 0.89	25.49 ± 1.31	9.93 ± 2.54	16.35 ± 6.17
YRL45	22.68 ± 1.37	23.55 ± 0.86	24.65 ± 2.18	25.73 ± 2.25	10.55 ± 2.53	16.88 ± 5.48

Note: There was no significant difference (*p* > 0.05) in the same column (*n* = 11).

**Table 3:** Effect of *L. plantarum* YRL45 on liver, spleen and thymus coefficients of mice.

Groups	Liver Coefficient (mg/g)	Spleen Coefficient (mg/g)	Thymus Coefficient (mg/g)
Control	43.21 ± 6.47	3.83 ± 0.95	1.68 ± 0.25
YRL45	41.56 ± 8.06	3.84 ± 1.53	1.87 ± 0.21

Note: There was no significant difference (*p* > 0.05) in the same column (*n* = 11).

## Data Availability

The data presented in this study are available on request from the corresponding author.
